# Association of *CAPN10* gene (rs3842570) polymorphism with the type 2 diabetes mellitus among the population of Noakhali region in Bangladesh: a case-control study

**DOI:** 10.5808/gi.23023

**Published:** 2023-09-27

**Authors:** Munia Sultana, Md. Mafizul Islam, Md. Murad Hossain, Md. Anisur Rahman, Shuvo Chandra Das, Dhirendra Nath Barman, Farhana Siddiqi Mitu, Shipan Das Gupta

**Affiliations:** Department of Biotechnology and Genetic Engineering, Noakhali Science and Technology University, Noakhali 3814, Bangladesh

**Keywords:** Bangladesh, *CAPN10* gene, genotype, polymorphism, T2DM

## Abstract

Type 2 diabetes mellitus (T2DM) is a multifactorial, polygenic, and metabolically complicated disease. A large number of genes are responsible for the biogenesis of T2DM and calpain10 (*CAPN10*) is one of them. The association of numerous *CAPN10* genetic polymorphisms in the development of T2DM has been widely studied in different populations and noticed inconclusive results. The present study is an attempt to evaluate the plausible association of *CAPN10* polymorphism SNP-19 (rs3842570) with T2DM and T2DM-related anthropometric and metabolic traits in the Noakhali region of Bangladesh. This case-control study included 202 T2DM patients and 75 healthy individuals from different places in Noakhali. A significant association (p < 0.05) of SNP-19 with T2DM in co-dominant 2R/3R vs. 3R/3R (odds ratio [OR], 2.7; p=0.0014) and dominant (2R/3R) + (2R/2R) vs. 3R/3R (OR, 2.47; p=0.0011) genetic models was observed. High-risk allele 2R also showed a significant association with T2DM in the allelic model (OR, 1.67; p=0.0109). The genotypic frequency of SNP-19 variants showed consistency with Hardy-Weinberg equilibrium (p > 0.05). Additionally, SNP-19 genetic variants showed potential associations with the anthropometric and metabolic traits of T2DM patients in terms of body mass index, systolic blood pressure, diastolic blood pressure, total cholesterol, and triglycerides. Our approach identifies the 2R/3R genotype of SNP-19 as a significant risk factor for biogenesis of T2DM in the Noakhali population. Furthermore, a large-scale study could be instrumental to correlate this finding in overall Bangladeshi population.

## Introduction

Type 2 diabetes mellitus (T2DM) is a complex metabolic disease characterized by reduced peripheral tissue response to insulin (insulin resistance) coupled with inadequate insulin secretion due to β-cell dysfunction [1]. Most of the T2DM patients have been affected by this disease in their middle age [2]. T2DM can be considered as a global epidemic, as its prevalence has been spread throughout the world.

The International Diabetes Federation (IDF) estimated that 536.6 million people globally (10.5%) had T2DM in 2021, and the incidence is expected to reach 783.2 million (12.2%) by the year 2045 [3]. Compared to other major regions of the world, South-East Asia has seen the fastest increase in the prevalence of T2DM [4] where almost 90% to 95% of all diagnosed diabetes cases in this region are T2DM [5,6]. Alike with other south-east population, a large portion of the Bangladeshi population is also affected by T2DM. According to a comprehensive evaluation of previous studies (from 1994 to 2013), between 4.5% to 35% of people in Bangladesh are reportedly suffering from T2DM [7]. In Bangladesh's rural areas, over 20%–30% of adults have abnormal fasting blood sugar or impaired glucose tolerance and by the year 2030, it is anticipated that the prevalence of diabetes (mainly T2DM) will increase to 24%–34% [8]. T2DM causes several complications to the heart, kidneys, eyes, and other organs. The risk of T2DM is determined by a combination of non-changeable (age, family history of diabetes, and race/ethnicity) and changeable (obesity, smoking, sedentary lifestyle, diet, physical and emotional stress) factors linked to rapid urbanization and changing lifestyle [9].

Several environmental, multifactorial, and genetic causes of T2DM have been identified [1], and case-control and family studies have demonstrated a genetic predisposition to the disease [10,11]. Many genes related to the susceptibility to T2DM have been discovered by genome-wide association studies in different populations [10,12,13]. Calpain 10 (*CAPN10*) is one of the many genes linked to an increased risk of developing T2DM [14-17]. *CAPN10* is a ubiquitously expressed protease located on chromosome 2q37.3 [18]. The *CAPN10* gene consists of 15 exons, spanning a length of 31 kb, and encodes a calcium-dependent, non-lysosomal intracellular protease comprising 672 amino acids [19]. Research suggests that the *CAPN10* protein plays a role in actin rearrangement in pancreatic INS-1 cells (insulinoma cell line), which is crucial for insulin release in response to glucose [20]. Moreover, *CAPN10* protein controls both insulin-mediated glucose metabolism and insulin secretion [17,21,22]. Genetic and functional data implies that *CAPN10* also plays a crucial role in insulin resistance and intermediate phenotypes, which may provide an explanation for how it affects the development of T2DM [23]. In addition, the tissues of human heart, kidney, brain, pancreas, lungs, and liver are found to exhibit extremely high levels of *CAPN10* mRNA expression with different mRNA isoforms [22,24,25]. In nondiabetic patients, *CAPN10* mRNA levels are found to be associated with the increased insulin secretion, but this correlation is absent in diabetic patients [21].

Numerous polymorphisms namely SNP-110 (rs7607759), SNP-63 (rs5030952), SNP-56 (rs2975762), SNP-19 (rs3842570), SNP-43 (rs3792267), SNP-44 (rs2975760), etc. have been identified in *CAPN10* for their association with various disease. Among the *CAPN10* gene polymorphisms, SNP-19 (rs3842570), SNP-43 (rs3792267), SNP-44 (rs2975760), and SNP-63 (rs5030952) are the variants largely known for their association with T2DM risk [26-30]. However, the most studied *CAPN10* polymorphism concerned with T2DM is SNP-19 (rs3842570), which is a 32 base pairs indel polymorphism located in intron 6 [31]. Despite its location in the intronic region, which does not directly affect the amino acid structure of the protein, it is likely to influence gene expression or employ alternative splicing mechanisms [25]. SNP-19 profoundly affects insulin sensitivity as determined by the altered transcriptional regulation, which may increase the likelihood of developing diabetes [32]. Association of *CAPN10* SNP-19 with T2DM has been observed in several ethnic groups such as Mexican Mestizos [31], south Indians [33], Javanese [34], Koreans [35], Egyptians [30], Tunisian Arabs [19], and Spanish [36]. It is also evident that SNP-19 is associated with several metabolic traits such as obesity [19,32,37-41], high total cholesterol (TC) [29,30,42], high triglycerides (TG) [37,43], systolic blood pressure (SBP), and diastolic blood pressure (DBP) [35] in T2DM patients.

To the best of our knowledge, no association study of *CAPN10* SNP-19 variants with T2DM was performed previously in the Bangladeshi population. Hence, this study aimed at filling this gap by evaluating the plausible association of SNP-19, with the development of T2DM risk among the people of the Noakhali region in Bangladesh. Additionally, we examined the association of *CAPN10* SNP-19 with anthropometric and metabolic traits among T2DM.

## Methods

### Ethical statement

The study protocol and questionnaire were approved by the Ethical Clearance Committee, Faculty of Science, of the Noakhali Science and Technology University. Furthermore, both the cases and controls gave their signed consent to take part in this study after a briefing session.

### Study population and data collection

A total of 277 individuals, including 202 T2DM patients and 75 healthy controls of comparable aged group (above 30 years), were considered for this study. Blood samples (3 mL) of the T2DM patients and control group were collected from Al-Haj Sirajul Islam Diabetic and General Hospital, Maijdee, Noakhali, Chittagong, and from different places in Noakhali, respectively. All subjects were born in the greater Noakhali region of Bangladesh. Data were collected through face-to-face interviews using a systematic questionnaire that encompassed socio-demographic information, including age, sex, occupation, education, marital status, weight, height, and blood pressure. Additionally, profiles of diabetes mellitus were captured, such as the duration of diabetes, age at onset, fasting plasma glucose level, 2-h postprandial blood glucose level, usage of any antidiabetic drugs, and the presence of any heart disease. Furthermore, factors such as addiction to alcohol/smoking, family history of diabetes/heart disease, and living area were also included in the questionnaire. Patients with gestational diabetes or any pathophysiological diseases such as cancer were excluded from this study. Body mass index (BMI) was calculated from weight and height, and blood pressure was measured for all the subjects before withdrawing the blood sample. The genetic analysis was executed in the Molecular Biology Laboratory, Department of Biotechnology and Genetic Engineering, Noakhali Science and Technology University, Bangladesh.

### Collection of blood sample and molecular analysis

Blood samples (3 mL) were collected by a phlebotomist through the puncture of veins from 202 T2DM patients and 75 controls in sterile tubes containing ethylenediaminetetraacetic acid (EDTA)-Na_2_. Collected blood samples were transferred into two separate micro-centrifuge tubes. One tube was stored at –20°C for genomic DNA extraction and another one was centrifuged at 12,000 rpm for 12 min for isolation of blood plasma. The clear supernatant layer of plasma was transferred into a new autoclaved micro-centrifuge tube for further analyses. Genomic DNA was extracted from the blood samples by using FavorPrep Blood Genomic DNA Extraction Mini Kit (Favorgen Biotech Corp., Taiwan).

### Anthropometric and metabolic traits

Several anthropometric and metabolic traits of the T2DM patients such as BMI, SBP, DBP, TC, and TG level were determined for both patients’ and controls. The TC and TG levels were assessed using the spectrophotometric approach with commercially available kits (Human-GmbH, Wiesbaden, Germany). All the assays were performed in triplicate in order to minimize error.

### *CAPN10* genotyping

Genotyping of *CAPN10* SNP 19 (rs3842570) was determined through polymerase chain reaction (PCR). To amplify a partial fragment of the *CAPN10* gene harboring SNP-19, the following primer set was employed in the PCR; forward primer: 5ʹ-GTTTGGTTCTCTTCAGCGTGGAG-3ʹ and reverse primer: 5ʹ-CATGAACCCTGGCAGGGTCTAAG-3ʹ. PCR reactions were performed in 20 µL volume reaction mixtures using about 1 µL (5 ng/µL) of genomic DNA, 10 µL Master mix (2×) (Biolab, GoTaq, Promega, Madison, WI, USA), 7 µL of nuclease-free water (ddH_2_O), 1 µL of each primer. Products were amplified in the MiniAmp Plus thermocycler (Applied Biosystems, Waltham, MA, USA) for 35 cycles under the following conditions: denaturing at 95°C for 1 min, annealing at 53°C for 30 s and extension at 72°C for 1 min. The final extension step was for 5 min. Three percent agarose gel was used to separate the PCR products and the results were visualized under a UV trans-illuminator.

### Statistical analysis

The genotypes of both T2DM patients and controls were assessed for Hardy-Weinberg equilibrium (HWE) using gene-calc software (https://gene-calc.pl/hardy-weinberg-page). A significance level of p > 0.05 was applied in the analysis. Association analysis of the genotypes with both groups was determined using Medcalc Odd Ratio Calculator (https://www.medcalc.org/calc/odds_ratio.php). To assess the significance of the results, various genetic models were examined, including the co-dominant model (comparing 3R/3R vs. 2R/3R and 3R/3R vs. 2R/2R), the dominant model ((2R/3R) + (2R/2R) vs. 3R/3R), the recessive model ((3R/3R) + (2R/3R) vs. 2R/2R), and the allele model (comparing 3R vs. 2R). The level of significance for the analysis was set at 5% (p < 0.05). Odds ratio (ORs) and their corresponding 95% confidence interval (95% CI) were calculated for every model. Unless otherwise specified, an OR of one means that both the patient and the control groups are equally likely to encounter the event. Conversely, an OR greater than one indicates that the event is more likely to occur in the patient group, suggesting it as a risk factor for the disease. On the other hand, if the OR is less than one, it implies that the event is less likely to occur in the patient group, indicating it as a preventive factor [44].

To evaluate the association between the anthropometric and metabolic traits of both the T2DM patients and control groups based on genotypes, a two-tailed t-test was conducted using the Microsoft Office 11 Excel tool. The rationale for selecting a two-tailed t-test was to account for the possibility of both positive and negative deviations from the mean, thereby encompassing the entire range of potential differences between the groups.

## Results

### Characteristics of the participants

A total of 202 patients with T2DM and 75 healthy individuals were enrolled in this study. The majority of the T2DM patients were female (63.9%). In contrast, most of the control subjects were male (64.0%). Data of anthropometric and metabolic characteristics of the study groups were expressed as mean ± standard deviation (SD) (Table 1). A significant difference in the mean values of SBP, DBP, TC, and TG were observed between the group with T2DM and control subjects. In T2DM patients, a high level of TC was found in 44.5% of cases, while elevated TG was present in 61.9%. Furthermore, cardiovascular complications, including diabetic cardiomyopathy, heart attack, stroke, peripheral arterial disease, and heart valve complications, were identified in 41.1% of individuals within the T2DM group.

### Influences of the SNP-19 in T2DM

**Fig. 1.**
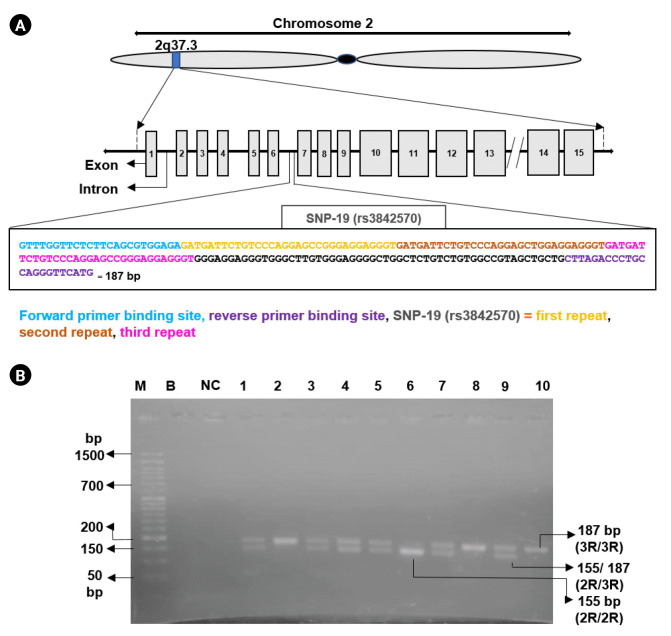
(A) Physical map of calpain 10 (*CAPN10*) rs3842570 with primer binding sites. The *CAPN10* gene is located on the human chromosome 2, at position 2q37.3. *CAPN10* gene has 15 exons (silver boxes). Introns are indicated in the linear line. The intron-6 carries SNP rs3842570. Nucleotides marked in blue color indicate the binding site of forward primer. Nucleotides in purple color denote the binding site of reverse primer. The orange, dark orange, and pink color nucleotides indicates first, second and third repeats of SNP-19 (rs3842570) respectively. (B) Agarose gel electrophoresis (3%) showing polymerase chain reaction products of SNP-19: lane 6 represents homozygous (2R/2R, 155 bp) of two repeats of 32 bp; lane 2, 8, and 10 represents homozygous (3R/3R, 187 bp) of three repeats of 32 bp and lanes 1, 3, 4, 5, 7, and 9 represent heterozygous genotype (2R/3R, 155 bp and 187 bp). Here, M denotes DNA marker (50 bp), B indicates blank, and NC denotes negative control.

The *CAPN10* gene is comprised of 15 exons. SNP-19 (rs3842570) is located within the intron sequence between exon 6 and exon 7 of *CAPN10* (Fig. 1A). This particular SNP-19 of *CAPN10* exhibits two allelic variations. Allele 1 consists of three repeats of a 32 bp sequence, while allele 2 consists of two repeats of the same 32 bp sequence. The binding sites of the designed primers, which encompass SNP-19, are illustrated in Fig. 1A.

A successful amplification of the targeted *CAPN10* fragment carrying rs3842570 polymorphism was obtained by PCR for both patients and control samples (Fig. 1B). Various patterns of DNA bands were observed for each genotype. Allele 1 (two repeats [2R] of 32 bp) was detected as a fragment of 155 bp and allele 2 (three repeats [3R] of 32 bp) was detected as a fragment of 187 bp, respectively (Fig. 1).

HWE was evaluated for each genotype of T2DM patients and healthy controls. The genotypic distribution fit with HWE (p > 0.05) (Table 2).

To evaluate the impact of *CAPN10* (rs3842570) polymorphism on T2DM, the co-dominant, dominant, recessive, and allele models were tested. The frequency of genotypes (3R/3R, 2R/3R, and 2R/2R) for the patients’ group was (28.2%, 56.9%, and 14.9%), and for the control group it was (49.3%, 38.7%, and 12.0%). Moreover, the difference between the two groups was statistically significant for the 2R/3R genotype (p = 0.0014) and the result wasn’t significant in case of 2R/2R. The rs3842570 of the *CAPN10* gene also proved significant association with T2DM risk in the dominant model and in the allelic model (p = 0.0011 and p = 0.0109), respectively (Table 3). Recessive model showed no significant association (p = 0.5451) with T2DM. The frequency of (2R/3R) + (2R/2R) in the dominant model was higher in-patient group (71.8%) and in the recessive model frequency of 2R/2R was 14.9% in the patient group. Both the frequencies were greater than their control group.

The frequency of (3R and 2R) alleles was 56.7% and 43.3% in the patients’ group; whereas, in the control group it was 68.7% and 31.3%, respectively. The OR for each model (co-dominant, dominant, recessive, and allele models) was evaluated also (OR, 2.57; OR, 2.47; OR, 2.16; OR, 1.67).

### Association of rs3842570 with T2DM patients according to anthropometric and metabolic traits

**Fig. 2.**
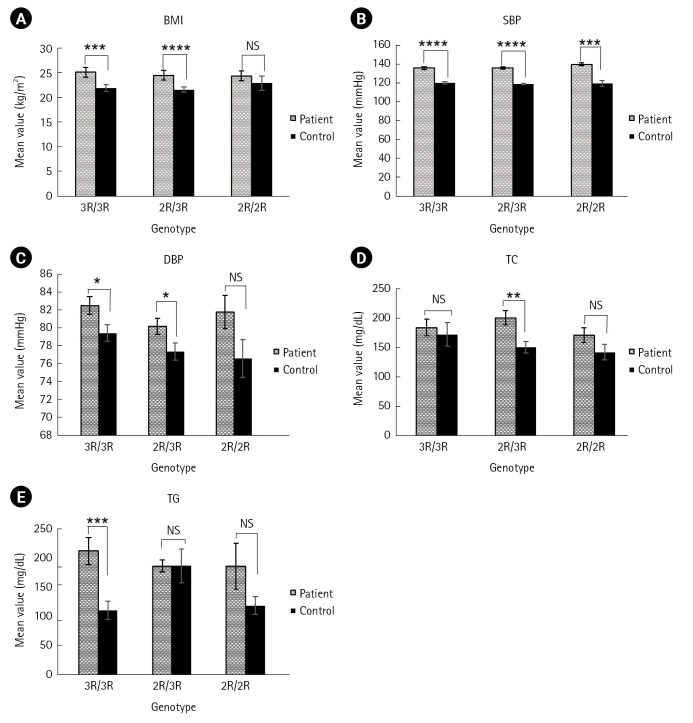
Analysis of anthropometric and metabolic traits according to genotype among the patients and controls: (A) body mass index (BMI, kg/m^2^) vs. genotype, (B) systolic blood pressure (SBP, mmHg) vs. genotype, (C) diastolic blood pressure (DBP, mmHg) vs. genotype, (D) total cholesterol (TC, mg/dL) vs. genotype, (E) triglycerides (TG, mg/dL) vs. genotype. ^*^p ≤ 0.05, ^**^p ≤ 0.005, ^***^p ≤ 0.0005, ^****^p ≤ 0.00005.

Association of the anthropometric and metabolic traits between the T2DM patients’ and controls’ group based on genotypes is shown in Table 4. The 2R/3R genotype showed significant differences between the patients and controls in terms of BMI (p = 4.42E-05), SBP (p = 6.66E-17), DBP (p = 0.034986), TC (p = 0.001532), respectively and no significant result was found in case of TG (Fig. 2). Along with the 2R/3R genotype, other genotypes also revealed significant differences between the studied groups in terms of the anthropometric and metabolic traits such as 3R/3R genotype in the BMI (p = 0.000287), SBP (p = 3.99E-10), DBP (p = 0.028086), TG (p = 0.000223), and 2R/2R genotype exhibits significant outcome with respect to SBP (p = 0.000411) only.

## Discussion

The majority of T2DM patients in our study (98.01% of the population) were above the age of 35. Age-related declines in insulin sensitivity and inadequate beta cell function compensate for rising insulin resistance are the primary contributing causes for this [45]. Likewise a study in Chittagong, Bangladesh also observed a large percentage (almost over 80%) of their study population were above the of 35 [46]. In our study, higher BMI (overweight or obesity) was identified in about 51.98% of the T2DM subjects, which aligns closely with a study conducted on the Indian population where the prevalence was 42% [47]. Another study performed on the Bangladeshi population found the prevalence of overweight or obesity in 67% of the population [46].

The association between *CAPN10* polymorphism and the risk of T2DM has been established in certain populations [16,19,25,30,31,33,34,35,48]. However, this relationship has not been consistently observed across all populations investigated [49-52]. For example, strong associations between *CAPN10* polymorphisms and T2DM have been found in Mexican Mestizos, south Indians, Javanese, Koreans, Egyptians, Tunisian Arabs, and Spanish population. Conversely, studies conducted in the Iraqi and Navi Mumbai populations of India did not reveal a robust correlation with T2DM [51,52]. In this genetic association study, we evaluated whether the *CAPN10* SNP-19 (rs3842570) was associated with the risk of T2DM among the Noakhali region of Bangladeshi population. Our results revealed that the 2R/3R genotypic frequency was higher in T2DM subjects compared to the nondiabetic individuals (56.9% vs. 38.7%) and statistically significant difference was seen between the two groups (p = 0.0014) (Table 3). These findings are in agreement with the result of Mexican Mestizos populations where patients' 2R/3R genotype (50.7%) was found greater than the controls’ group 42.8%) [31]. In our study population, the frequency of the risk allele 2R was found higher in the patient group (43.3%) than in the control group (31.3%) (Table 3). The genotyping distributions were in the line of HWE (p > 0.05) in all cases, both in patients (*χ*^2^ = 5.13, p = 0.07701) and in controls (*χ*^2^ = 0.77153, p = 0.67993) (Table 2). Logistic regression analysis was performed for 2R/3R and 2R/2R genotypes with 3R/3R as a reference genotype (OR, 1). In the co-dominant model, the 2R/3R genotype significantly conferred 2.57 times (OR, 2.57; 95% CI, 1.44 to 4.60; p = 0.0014) higher risk for the development of T2DM compared to the 3R/3R genotype (Table 3). The 2R/2R genotype also showed higher risk for T2DM than the 3R/3R genotype carriers but no significant association was found (OR, 2.16; p = 0.0758). A significant association in the dominant model was observed, where (2R/3R) + (2R/2R) carriers conferred 2.47-fold (OR, 2.47; 95% CI, 1.43 to 4.28; p = 0.0011) higher risk for T2DM in compared with 3R/3R genotype (Table 3). However, the association in the recessive model was not significant in our study. Unlike our findings a research with patients from the Turkish community showed a significantly increased risk of T2DM due to the 2R/2R genotype of the genetic polymorphism *CAPN10* (SNP-19) [53]. Our result was partially consistent with a prior study (univariate analysis) on Tunisian Arabs, which found that the 3R/2R had progressively higher T2DM risk (OR, 1.35; 95% CI, 1.08 to 1.68; p = 0.008) in addition to 2R/2R (OR, 1.61; 95% CI, 1.20 to 2.18; p = 0.002) [54] but multivariate analysis on Tunisian Arabs didn’t support our findings where homozygous SNP-19 2R/2R found to be responsible for T2DM risk (p = 0.044; OR, 1.40; 95% CI, 1.01 to 1.93) [54]. Our findings were found comparable to the SNP-43, SNP-19, and SNP-63 diplotype (112/121), which conveys that the 2R/3R genotype at SNP-19 (allele 1 = 2R, allele 2 = 3R) was associated with 2.8, 2.55 and 4.97 fold higher T2DM risk in the Mexican-American, Finnish and German populations, respectively [25]. Also in an Egyptian and Korean population the most frequent diplotype 111/121 (SNP-43, SNP-19, and SNP-63) with the presence of 2R/3R genotype of SNP-19 was found to be associated with increased risk of T2DM [30,35]. Further in the allelic model, the 2R allele was observed to confer 1.67 times higher risk for T2DM compared to the 3R allele and the result was statistically significant (OR, 1.67; 95% CI, 1.13 to 2.49; p = 0.0109). Similarly, one Tunisian (Arab) study found an association between the 2R allele and T2DM (p = 0.007; OR, 1.15) [54]. 2R allele of SNP-19 was also associated with increased risk of T2DM in Finnish population [22].

Our study revealed the association of certain metabolic and anthropometric traits such as BMI, SBP, DBP, TC, and TG with the genotypes of *CAPN10* rs3842570 between the group of T2DM patients’ and controls’ (Fig. 2). In this study, BMI was found significantly higher in patients with 2R/3R genotype compared to other two genotypes (Table 4). A similar study performed on Ciudad Juarez, Mexico population discovered a link between the 2R/3R genotype and a higher BMI (p = 0.037) in T2DM patient [55]. Our result partially supports the result of a study carried out on Turkish people where 3R/3R found to be related with a significantly increased BMI, but only in male diabetic patients [40] and some study on Brahmin Indians, Tunisian Arab and Japanese, also revealed that T2DM patients had a risk of increasing BMI that is related with the 3R/3R genotype (p = 0.007, p = 0.003, and p = 0.003) [32,38,54]. Subject with genotype 2R/3R had the highest mean of BMI in T2DM group among Javanese people but no significant difference was found both in-patient and control group [34]. In this study, the 3R/3R genotype also showed significant differences (p = 0.000287) in the patients for BMI when compared with control group (Table 4). In terms of TC and SBP, the 2R/3R genotype was found significantly higher in patients compared to controls in our study (p = 0.001532 and p = 6.66E-17). Our result agrees with a study in the Korean population, where T2DM patients with 111/121 haplotype combination (defined by SNP-43, SNP-19, and SNP-63) containing the 2R/3R (allele 1 = 2R, allele 2 = 3R) genotype showed significantly higher level of TC (p = 0.034) and SBP (p = 0.041) [35]. Again, another study on the Chinese population revealed that haplotype combination 112/121 (SNP-43, SNP-19, and SNP-63) might be a risk factor for increased serum cholesterol in T2DM patients [42]. On the contrary, among the Gaza strip patients with the 121/221 haplotype combination carrying the 3R/3R genotype had the highest TC levels (p = 0.005) [29]. We also found that 2R/3R and 3R/3R genotype had significant differences in the patients compared to controls for DBP and this is partially consistent with the result found in the Korean population [35].

Our results revealed that TG had significant mean differences in patients with 3R/3R genotype (p = 0.000223) which is comparable to the study conducted on the Korean population where 111/121 diplotype showed significantly higher TG concentration [35]. Moreover, our outcome did not support the result of the Mexican Mestizos population where TG were significantly higher in patients with 2R/2R and 2R/3R genotypes [43]. The 2R/3R genotype showed comparatively more significant differences than the other genotypes (3R/3R and 2R/2R) in terms of BMI, SBP, and TC, respectively (Fig. 2). Discrepancies of our result from other population studies might be attributed to changes in-patient characteristics, data presentation, sample size, and statistical power. However, as our study only looked at one SNP, it's possible that other *CAPN10* SNPs played a role in the pathogenesis of T2DM among the Noakhali region of Bangladeshi population.

The present study indicates a significant association between 2R/3R for the SNP-19 of *CAPN10* and T2DM. Our findings demonstrated that the 2R/3R genotype of *CAPN10* SNP-19 (rs3842570) might be a significant risk factor for T2DM patients and T2DM-related metabolic traits among the people of greater Noakhali region. This study may help us develop more effective personalized T2DM treatment plans. Further, a larger-scale investigation needs to be conducted to reach to a conclusive remark for the potential contribution of *CAPN10* SNP-19 in the pathogenesis of T2DM in our studied population.

## Figures and Tables

**Table 1.** Demographic, anthropometric and metabolic traits of patients with T2DM and controls

Trait	T2DM (n = 202)	Control (n = 75)	Normal range
Sex (M/F), n (%)	73/129 (36.1/63.9)	48/27 (64.0/36.0)	NA
Age (y)	55.51 ± 10.55	40.02 ± 7.59	NA
BMI (kg/m^2^)	24.94 ± 3.64	22.34 ± 3.21	18.5–24.9
SBP (mmHg)	135.09 ± 16.58	118.50 ± 4.39	120–129
DBP (mmHg)	81.10 ± 9.07	78.52 ± 5.12	80–84
TC (mg/dL)	191.40 ± 79.99	156.98 ± 57.22	<200
TG (mg/dL)	193.96 ± 104.99	151.21 ± 114.69	>150

Values are presented as mean ± SD. T2DM, type 2 diabetes mellitus; NA, not available; BMI, body mass index; SBP, systolic blood pressure; DBP, diastolic blood pressure; TC, total cholesterol; TG, triglycerides; SD, standard deviation.

**Table 2.** Distribution of genotypes of *CAPN10* SNP-19 (rs3842570) and HWE test

SNP-19 (rs3842570)	T2DM			Control		
	No. (%) (n = 202)	χ^2^	p-value	No. (%) (n = 75)	χ^2^	p-value
3R/3R	57 (28.2)	5.13	0.07701^a^	37 (49.3)	0.77153	0.67993^a^
2R/3R	115 (56.9)			29 (38.7)		
2R/2R	30 (14.9)			9 (12.0)		

*CAPN10*, calpain10; HWE, Hardy-Weinberg equilibrium; T2DM, type 2 diabetes mellitus.

a p > 0.05 considered as consistent with HWE.

**Table 3.** Association of *CAPN10* SNP-19 (rs3792267) with T2DM cases and healthy controls in terms of genotypes and alleles

SNP-19 (rs3842570)	T2DM (n = 202)	Controls (n = 75)	OR (95% CI)	p-value
Co-dominant model				
3R/3R	57 (28.2)	37 (49.3)	1	-
2R/3R	115 (56.9)	29 (38.7)	2.57 (1.44–4.60)	0.0014^*^
2R/2R	30 (14.9)	9 (12.0)	2.16 (0.92–5.07)	0.0758
Dominant model				
3R/3R	57 (28.2)	37 (49.3)	1	
(2R/3R) + (2R/2R)	145 (71.8)	38 (50.7)	2.47 (1.43–4.28)	0.0011^*^
Recessive model				
(3R/3R) + (2R/3R)	172 (85.1)	66 (88.0)	1	-
2R/2R	30 (14.9)	9 (12.0)	1.27 (0.58–2.84)	0.5451
Allele model				
3R allele	229 (56.7)	103 (68.7)		
2R allele	175 (43.3)	47 (31.3)	1.67 (1.13–2.49)	0.0109^*^

*CAPN10*, calpain10; T2DM, type 2 diabetes mellitus; OR, odds ratio; 95% CI, 95% confidence interval.

* Statistically significant result (p < 0.05).

**Table 4.** Association of anthropometric and metabolic traits in each genotype of the T2DM patients and controls

Trait	3R/3R		2R/3R		2R/2R	
	Patient	Control	Patient	Control	Patient	Control
BMI	25.36 ± 3.08	22.12 ± 2.96	24.71 ± 3.82	21.80 ± 2.68	24.63 ± 3.26	23.13 ± 3.59
	p = 0.000287^***^		p = 4.42E-05^****^		p = 0.374236	
SBP	134.29 ± 13.80	118.95 ± 4.59	134.65±16.88	117.63 ± 3.5	138.33 ± 20.27	118.33 ± 7.53
	p = 3.99E-10^****^		p = 6.66E-17^****^		p = 0.000411^***^	
DBP	82.55 ± 7.26	79.47 ± 4.05	80.22 ± 9.54	77.4 ± 4.81	81.83 ± 10.13	76.67 ± 5.16
	p = 0.028086^*^		p = 0.034986^*^		p = 0.086673	
TC	184.06 ± 74.17	172.30 ± 76.78	200.34 ± 88.76	150.69 ± 46.81	170.85 ± 46.23	142.51 ± 29.18
	p = 0.629746		p = 0.001532^**^		p = 0.140732	
TG	212.12 ± 125.22	110.46 ± 64.67	186.56 ± 79.53	186.86 ± 140.86	185.95 ± 146.69	118.64 ± 34.04
	p = 0.000223^***^		p = 0.992137		p = 0.129066	

Values are presented as mean ± SD. T2DM, type 2 diabetes mellitus; BMI, body mass index; SBP, systolic blood pressure; DBP, diastolic blood pressure; TC, total cholesterol; TG, triglycerides; SD, standard deviation.

* p ≤ 0.05,

** p ≤ 0.005,

*** p ≤ 0.0005,

**** p ≤ 0.00005.
